# Optimal Papanicolaou Smear Conditions for Manual Microdissection of Single Target Cells

**DOI:** 10.3390/microorganisms11112700

**Published:** 2023-11-03

**Authors:** Kaori Okayama, Mao Kakinuma, Saki Tajima, Norika Nagasawa, Yasuyoshi Ishii, Mizue Oda, Hirokazu Kimura, Mitsuaki Okodo

**Affiliations:** 1 Department of Medical Technology, Faculty of Health Sciences, Gunma Paz University, 1-7-1 Tonyamachi, Takasaki-shi 370-0006, Gunma, Japan; 2 Genki Plaza Medical Center for Health Care, 3-6-5 Iidabashi, Chiyoda-ku, Tokyo 102-0072, Japan; 3 Department of Medical Technology, Faculty of Health Sciences, Kyorin University, 5-4-1 Shimorenjaku, Mitaka-shi, Tokyo 181-8621, Japan

**Keywords:** manual microdissection, human papillomavirus, Papanicolaou smear, PCR-based HPV testing

## Abstract

This study aimed to investigate the optimal conditions for Papanicolaou (Pap) smear to increase the success rate of target cell isolation through manual microdissection (MMD) and prevent cell spread. Pap smears were prepared using an HPV42-positive SurePath™ liquid-based cytology case, and 46 and 50 koilocytes were used in wet and dried Pap smears, respectively, to verify the success rate of target cell isolation using MMD based on the HPV detection rate. During MMD, the microscopic examination of both specimens revealed that cells in dried smears could be easily identified; however, cell debris remained in the surrounding area after MMD. Although it was difficult to observe cells in wet smears, there was no cell debris. When the needle tip was immersed in DNA lysate after cell isolation through MMD, a difference in cell solubility was found between dry and wet smears. HPV42 was detected in 94.7% and 97.4% of dried and wet Pap smears, respectively, via polymerase chain reaction genotyping using lysed cell solution; the detection rates were not significantly different. The isolation of target cells from wet Pap smears using MMD reduced the risk of contamination and increased the success rate of HPV detection. This study might facilitate the identification of new CIN-derived HPV-infected cells using MMD with wet Pap smears.

## 1. Introduction

Human papillomavirus (HPV) is strongly associated with the development of cervical intraepithelial neoplasia (CIN) [1,2]; therefore, HPV testing is the primary screening method for cervical cancer [3]. Women with a positive HPV test are triaged using reflex cytology to reduce cancer incidence and avoid unnecessary colposcopy tests [4,5]. However, triage using various molecular biological markers instead of cytology is recommended because the cytologically detected absence of intraepithelial lesion malignancy helps identify women who need this referral [6]. However, cytology, which is easily performed in clinical practices at no additional cost, remains the most effective triage method until reliable molecular biological markers are identified.

False-negative cytology results are commonly obtained because of several factors, including sampling and screening errors caused by the misinterpretation of cells on smears [7]; however, HPV-infected cells of CIN origin possibly present in the smear are usually not morphologically identified. Since most cervical lesions are caused by HPV, new lesion-derived HPV-infected cells must be investigated to increase the sensitivity of cytology for HPV detection.

We previously reported the successful detection of HPV in samples obtained through manual microdissection (MMD) of single-cell koilocytes [8] and multinucleated cells [9] on dried Papanicolaou (Pap) smears and confirmed that each cell is infected with a specific genotype. However, some issues were noted in the study: the success rate of isolating target cells on dried Pap smears through MMD was approximately 50% [8,9], and minor contamination due to the dispersion of cell fragments was observed [10].

This study aimed to determine the optimal Pap smear conditions to increase the success rate of target cell isolation using MMD and prevent cell spread. Lesion-derived HPV-infected cells were isolated using MMD in wet and dried Pap smears to demonstrate the differences in conditions.

## 2. Materials and Methods

### 2.1. Clinical Sample

A specimen was selected from the sample archive of the Genki Plaza Medical Center for Health Care, Tokyo, Japan, in which the infection of a single HPV type was confirmed using SurePath™ liquid-based cytology (LBC; Becton Dickinson and Company, Franklin Lakes, NJ, USA) cell suspension and koilocytes were identified in the LBC specimen. Of the specimens in which koilocytes were detected, one specimen that was infected with a single HPV type, as detected using uniplex E6/E7 polymerase chain reaction (PCR) method, and had a sufficient residual sample was used as a sample. This sample was HPV42-positive and identified as a low-grade squamous intraepithelial lesion based on cytological results and CIN grade 1 based on biopsy histology results.

To validate the success rate of target cell isolation using MMD for wet and dried Pap smears, HPV-infected koilocytes with a high viral load [11,12] were used as target cells for PCR-based HPV genotyping (detection limit of 100 copies), which helped exclude PCR false-negative cells because of low viral loads. If HPV was detected in a sample from which koilocytes were isolated using MMD, the isolation was considered successful.

### 2.2. Ethical Approval

This sample was collected after obtaining written informed consent from the patient. The Ethics Committee on Human Research of Kyorin University and Gunma Paz University approved the study protocol (2023–1, PAZ18-37), which was implemented in accordance with the approved guidelines.

### 2.3. MMD of Target Cells

Each cell pellet of the residual LBC sample was mounted as a single thin layer on a microscope slide and fixed with 95% ethanol. One cytotechnologist reviewed the Pap smear slides, and certain koilocytes were selected for sampling. In the targeted koilocytes, 46 and 50 cells were found in the dried and wet Pap smears, respectively. The selected cells were photographed individually. Thereafter, the slides were soaked in xylene to remove the cover slip. Xylene was washed away (15 min, 3 times) with 100% ethanol (10 min, 3 times). Subsequently, under a microscope, the selected cells were collected by gently picking up the edge of the cell using the tip of a 27-G injection needle (NN-2738R, TERUMO) [10]. In dried Pap smears, which were air-dried after immersion in 100% ethanol, single cells were scrapped off with the needle tip. Meanwhile, in wet Pap smears that were kept wet with 100% ethanol, the single cells were peeled off at the needle tip and floated in liquid, and the single cells were then caught using the side of the needle. The cells attached to the needle point were precisely transferred to each tube containing an alkaline lysis solution, and they were moved back and forth 10–20 times from side to side and left to rest for 1 min. After MMD, the disappearance of cells was visually confirmed using a 3-in-1 digital microscope (300,000 pixels) (Leanking, Tokyo, Japan), and needle tip photographs were taken.

### 2.4. HPV Genotyping

The single cell was lysed with 50 μL of an alkaline lysis solution (25 mM NaOH and 0.2 mM ethylenediamine tetra-acetic acid, pH 12.0) for 10 min at 95 °C [13]. Then, the lysed cell solution was centrifuged at 13,200 rpm for 1 min and directly used as the DNA template. For HPV genotyping, a uniplex E6/E7 PCR was used, as previously described by Okodo et al. [14]. This method can detect 39 mucosal HPV genotypes from as few as 100 viral copies, with no cross reactivity across all HPV genotypes. However, this PCR method may occasionally provide false-positive results. Therefore, to eliminate the possibility of DNA contamination, each round of PCR was performed with negative controls using DNase-free water (Takara Bio Inc., Shiga, Japan).

### 2.5. Statistical Analysis

Statistical analyses were performed using IBM SPSS Statistics version 25.0 (IBM Corp., Armonk, NY, USA). The success rate of target cell isolation using MMD on wet and dried Pap smears was compared on the basis of HPV detection rate, and significant differences were assessed using chi-squared tests. A *p*-value of <0.05 was considered statistically significant.

## 3. Results

**Figure 1:**
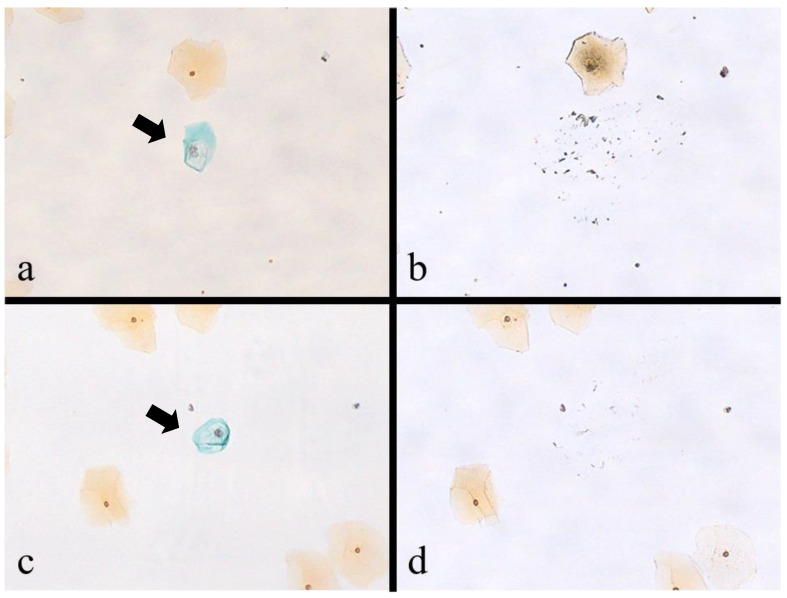
Before and after the isolation of single target cells from the Papanicolaou (Pap) smear. (**a**,**c**): Before the isolation of target cells using the manual microdissection (MMD) method. Arrows indicate koilocytes. (Pap stain, ×100); (**b**,**d**): after isolation of target cells using MMD (Pap stain, ×100). (**a**,**b**) Single target cells isolated via MMD from dried Pap smears. (**c**,**d**) Single target cells isolated via MMD from Pap smears wet with 100% ethanol.

**Figure 2:**
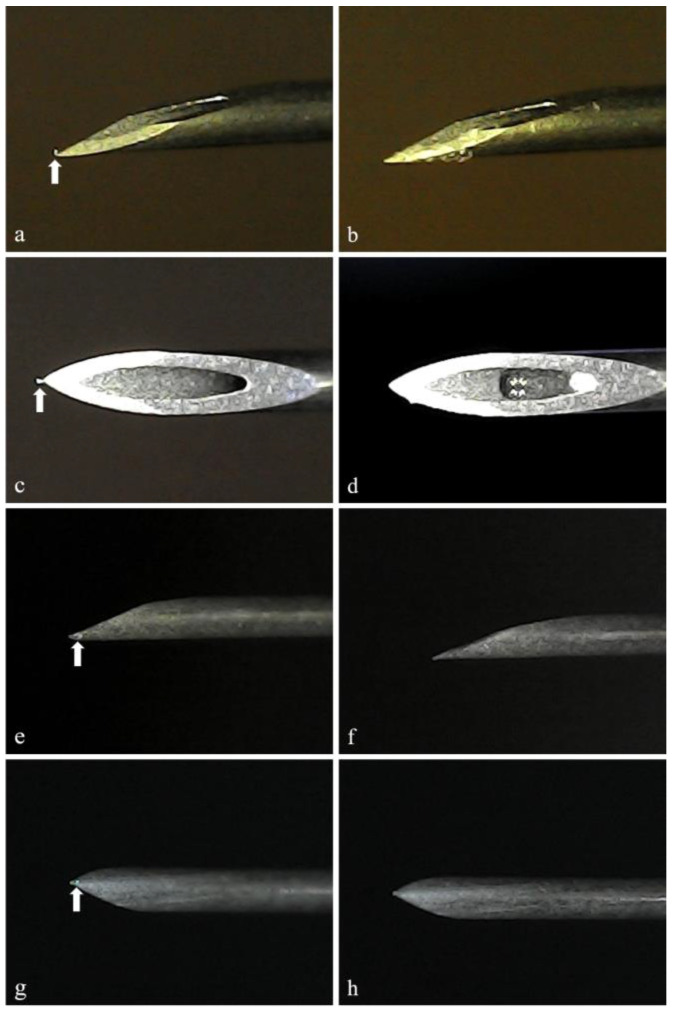
Needle tip before and after dipping it in an alkaline lysis solution. Single target cell (arrow) isolated using MMD is visible at the needle tip. (**a**,**c**): Cell isolated from dried Pap smear was present at the needle tip. (**e**,**g**): Cell isolated from wet Pap smear adhered to the sides of the needle. (**b**,**d**,**f**,**h**): Needle tip images after immersing in it an alkaline lysis solution. The target cell was completely lysed and disappeared. After heat treatment, this lysed cell solution was used as a DNA template.

The results of successful koilocyte isolation using MMD in dried Pap smears and ethanol-wet Pap smears are shown in Figure 1. Microscopic findings revealed that the cells in dried Pap smears could be easily identified during MMD (Figure 1a,b); however, cell fragments remained in the surrounding area after the MMD. Cells in wet Pap smears were difficult to observe (Figure 1c,d); however, no cell fragments remained after MMD. Needles after cell isolation but before immersion in an alkaline lysis solution are shown in Figure 2. Single target cells from the dried Pap smears tended to adhere to the needle tip (Figure 2a,c), whereas cells from the wet Pap smears tended to adhere to the side of the needle (Figure 2e,g). Target cells were excluded from the analysis if they were lost in the process of removing coverslips after clicking a photograph or if the needle tip came in contact with nontarget cells during MMD. Consequently, we obtained 38 target cells from dried and wet Pap smears.

When the needle tip was immersed in an alkaline lysis solution, the target cells isolated from dried Pap smears that were attached to the needle tip became invisible and were lysed 10 times. Conversely, cells from wet Pap smears remained on the needle tip even after the needle tip was moved >10 times; however, lysis was confirmed 20 times. Therefore, after the target cells at the needle tip were placed in an alkaline lysate, the needle tip was moved back and forth from side to side 10 times for dried Pap smears and 20 times for wet Pap smears and then allowed to stand still for 1 min. Photographs of cells from the dried (Figure 2b,d) and wet (Figure 2f,h) Pap smears attached to the needle tip after immersion in alkaline lysate are shown. The lysis of target cells isolated using MMD was confirmed by their disappearance in all samples.

Using the lysed cell solution as a DNA template, HPV42 was detected using PCR-based HPV genotyping in 94.7% (36/38 cells) of dried and 97.4% (37/38 cells) of wet Pap smears, showing no significant difference between them.

## 4. Discussion

No dried or wet smear conditions significantly affected the success rate of target cell isolation using MMD. Differences were observed in the MMDs of both smears. First, the visibility of target cells under the microscope and the ease of performing MMD were higher under dry conditions than under wet conditions. Second, as cell fragments persist on the glass slide after MMD, the likelihood of leaving cells on the specimen during MMD and inducing contamination due to scattering were higher in dried Pap smears [10] than in wet Pap smears. As the contamination of other target cells during MMD likely leads to the generation false-positive HPV results, wet smears should be preferred for MMD. To the best of our knowledge, this is the first study to report this finding.

Another novel finding of this study was the difference in the solubility of target cells attached to the needle tip isolated using MMD upon immersion in an alkaline lysis solution. To lyse the target cells, the needle tip must move back and forth and from side to side after MMD 10 and 20 times for cells in dried and wet Pap smears, respectively. This difference in the number of movements can be explained by the fact that dried cells are easily lysed because of their high affinity for the alkaline lysis solution, whereas wet cells have an affinity toward ethanol; therefore, developing the affinity of wet smears for the lysis solution requires more time. In addition, the needle tip should be immersed in the lysis buffer for a sufficient time after MMD to avoid false-negative results of HPV testing. In this study, the MMD success rate was twice that reported in previous reports [8,9], probably because the cells were not lysed for long previously. However, the underlying cause of this observation may depend not on the smear condition or MMD quality but on the amount of virus infecting each cell. HPV viral loads in cervical cancer cells in tissue have reported HPV16 at 9–630 copies [15]. Moreover, cells derived from CIN2 and 3 have HPV-DNA incorporated into their genome, and viral production is rare [16]. In this study, PCR-based HPV genotyping was performed with a detection limit of 100 copies, which could detect false-negative HPV results even in samples from which cells were successfully isolated using MMD. Thus, the use of MMD with high-viral-load koilocytes as target cells facilitated the appropriate evaluation of the success rate of cell isolation using MMD.

This study can enable the detection of HPV-DNA with high sensitivity in targeted single cells isolated using MMD. Further comprehensive analysis of atypical cells of unclear origin and the identification of new cytological signs of HPV infection of CIN origin will contribute to improving the accuracy of cytological testing.

## 5. Conclusions

Wet Pap smears are recommended for the isolation of target cells using MMD owing to the low risk of HPV-DNA contamination. In the future, new CIN-derived HPV-infected cells can be identified based on positive results of cells isolated using MMD that are of the same HPV genotype as the lesion tissue.

## Data Availability

Data sharing is not applicable to this article as no new data were created or analyzed in this study.
